# Seasonal fish larvae abundance and composition in seagrass habitats of coastal East Africa

**DOI:** 10.1038/s41598-024-62012-3

**Published:** 2024-05-16

**Authors:** Noah Ngisiange, Barnabas Tarimo, Lillian Daudi, Stephen Mwangi, Fadhili Malesa, Rushingisha George, Margareth S. Kyewalyanga, Martin Gullström, Melckzedeck Osore, James Mwaluma, Monika Winder

**Affiliations:** 1 Department of Ecology, Environment and Plant Sciences, Stockholm University, Stockholm, Sweden; 2 Directorate of Ocean and Coastal Systems, Kenya Marine and Fisheries Research Institute, Mombasa, Kenya; 3 Institute of Marine Sciences, University of Dar es Salaam, Zanzibar, Tanzania; 4 School of Aquatic Sciences and Fisheries Technology, University of Dar es Salaam, Zanzibar, Tanzania; 5 Tanzania Fisheries Research Institute, Dar-es-Salaam, Tanzania; 6 School of Natural Sciences, Technology and Environmental Studies, Södertörn University, Huddinge, Sweden

**Keywords:** Coastal East Africa, Seagrass habitat, Fish larvae, Abundance, Community composition, Seasonality, Ecology, Environmental sciences, Ocean sciences

## Abstract

Seagrass habitats play a major role in fisheries productivity through nursery functions and feeding grounds for diverse fish species. However, little is known about the seasonal distribution of fish larvae at large spatial scales in coastal East Africa. We investigated drivers of the seasonal fish larvae abundance and composition in seagrass habitats in Kenya and Tanzania. We found a high diversity of fish larvae (54 families) inhabiting seagrass habitats that differed between sites and seasons. Fish larvae abundance were highest in Kenya, particularly during the northeast monsoon season. Overall, total larval abundances per site were low, reaching less than 190 individuals/100 m^3^ in Kenya and less than 40 individuals/100 m^3^ in Tanzania, likely related to the low productivity and strong hydrodynamic processes in this region. Our data suggests that most of the fish spawn year-round in these tropical waters as we did not find strong seasonal patterns. All sites had a high relative abundance of larvae from demersal spawning fishes, indicating that many fish species move to coastal sites for spawning. Primary productivity and dissolved oxygen, driven by hydrodynamics conditions are positively related to fish larvae productivity both in Kenya and Tanzania. These findings indicate that the occurrence of both resident and transient fish larvae in seagrass meadows is driven by strong hydrodynamic and tidal processes that transport fish larvae across adjacent habitats.

## Introduction

Seagrass meadows are among the dominant shallow-water habitats in tropical coastal areas, providing numerous essential ecosystem functions, including the usage as nursery and feeding grounds for plentiful fish species^1,2^. The structural complexity of seagrass habitats is expected to provide shelter, food and protection from predation for fish larvae and juveniles^3–5^. The habitat complexity of seagrass meadows in combination with the relatively calm waters of nearshore areas is expected to support adult spawning and fish larval retention^6,7^, although some larval retention within habitats may also be affected by other factors such as spawning behaviour^8^. Because fish mortality during early life stages is a critical production constraint and a critical factor in ensuring the sustainability of fish stocks^9,10^, information on the distribution of fish larvae in coastal areas is necessary to provide an essential understanding of fish community biology and seasonal productivity patterns^11,12^. Yet, little is known about factors influencing the distribution of early life stages of fish, particularly in the coastal habitats of East Africa in the Western Indian Ocean (WIO), a region where fish are an essential source of food and income^13,14^. Seasonal variability of fish larvae community composition and abundance across shallow-water coastal habitats mainly focused on local area comparisons, covering a relatively narrow spatial scale^14–17^, which limits the understanding of how environmental conditions and ecological characteristics occurring at a larger scale (tens to hundreds of kilometres) influence the distribution of fish larvae.

Understanding the processes that drive fish larvae growth and survival is important for maintaining larval recruitment and fisheries productivity^18,19^. Only around 1% of the larvae that hatch from eggs survive to maturity^10,20^, mainly due to high starvation-induced mortality within the first days of hatching^21^, predation or death by natural causes due to complex environmental variability^22^. The early fish larval stages, including the egg-yolk and preflexion stages are free-floating and local hydrodynamic processes are therefore important for determining larval distribution and the environmental conditions they experience. At these early life stages, larvae can be transported on a large geographic scale or retained in certain areas by eddy formations that are, for example, driven by the configuration and location of islands. In addition, fish larvae may migrate vertically and distribute passively at a certain horizontal position^23^, contributing to larval retention in certain areas. At the late postflexion stage, fish larvae can actively move, orient and modify their dispersal through directional swimming behaviour^24,25^. For instance, during the flexion and postflexion development stages, some larvae species can swim up to 30 cm sec^-1^ horizontally and vertically^25^. At the late larval or juvenile stages, settlement to coastal areas typically occurs in species-specific patterns. Fish larval dispersal and settling are also related to the spawning mode of their parents^26^. Pelagic spawners, such as surgeonfishes (Acanthuridae), are characterized by many eggs and small-sized larvae released directly into the water column^18,27^. As a result, dispersal starts before the fish larvae phase, with a relatively long pelagic larvae duration and larger dispersal potential before settlement^28^. In contrast, demersal spawners, such as gobies (Gobiidae), attach their eggs to a substrate, producing relatively large fish larvae with a relatively short pelagic larval duration, short distance dispersal and overall fast larval development^29^.

Fish larvae abundance and community composition are also constrained by environmental factors, such as seasonal variations in current velocities, water temperature, salinity and oxygen concentration, that affect larval growth, survival and spawning time^30,31^, but these factors act differently depending on the area-specific context^4,32,33^. Environmental conditions trigger spawning and aggregation of fish larvae^34^, and are often synchronized with the lunar cycle. Fish larvae composition may consist of resident spawned larvae that travel short distances and are observed often year-round in tropical waters, while transient spawned larvae travel relatively long distances and often occur at specific times of the year. Families like Labridae, Scaridae, Serranidae, Acanthuridae, and Lutjanidae form resident spawning aggregations, although there may be differences between species^35^. Offshore spawning followed by a pelagic larval stage where larvae move to nearshore habitats and undergo a benthic stage is common in many fish species associated with seagrass beds^35^, such as the transient spawning aggregation taxa Sphyraenidae, Belonidae, Carangidae, Lethrinidae, and Gerreidae.

**Figure 1:**
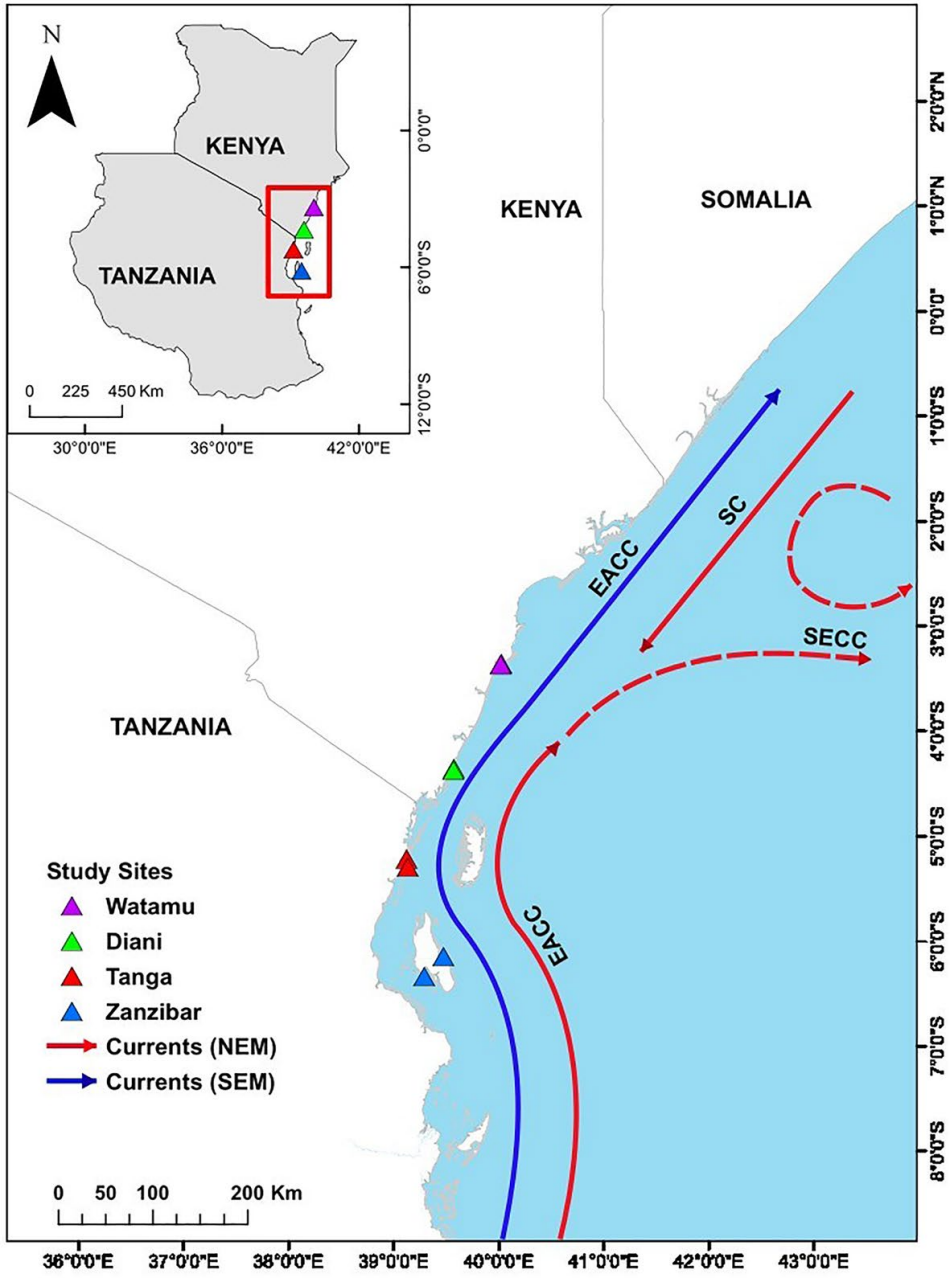
Map of the sampling area and dominating oceanic currents. The insert plot shows the study area along the East African coast and symbols the sampling locations. Sampling sites in Kenya are Watamu and Diani, and in Tanzania Tanga (mainland) and Zanzibar Island (Unguja, herein referred to as Zanzibar). Arrows indicate the dominating flow of the currents between different seasons i.e. SEM and NEM, i.e. the Somali Current (SC), South Equatorial Counter Current (SECC) and East African Coastal Current (EACC). The map was developed and generated using ArcGIS Desktop version 10.5^78^.

Larval supply is highly defined by coastal hydrodynamics that greatly influence larval transport. Currents as part of coastal hydrodynamics play an important role in determining material transport in the ocean, which also affects the processes of dispersion, retention and accumulation of fish larvae^36^. Along the East African coast, the horizontal current transport can take an eastern or northern direction^37^. This may disperse fish larvae between habitats or from offshore to inshore areas. Current patterns are influenced by monsoon seasons and the dispersal of larvae mostly occur during high tides with site-specific hydrodynamics that drive local changes in water currents and tides. The year-round, northward-flowing East African Coastal Current (EACC) largely drives oceanic conditions along the coastlines of Kenya and Tanzania^38^, but strong seasonality exists due to monsoon forcing (Fig. 1). Monsoon winds affect the extent of the EACC’s latitudinal range and velocity. During the southeast monsoon (SEM, April to October) season, the EACC is accelerated and extends its range northward, where it affects the entire Kenyan coastline^39^ through the northward current velocity (Nvel)^40^. During the northeast monsoon (NEM, November to March) season, the EACC weakens but does not reserve direction and positive vertical current velocities from the east develop, termed eastward current velocity (Evel)^40^. Although the monsoon winds and ocean currents are the predominant patterns, regional differences may cause variations in flow and circulation patterns, which may in turn affect fish larvae retention and dispersal patterns^41^.

In this study, we investigated the seasonal variability of fish larvae abundance and community composition across seagrass habitats in response to variability in environmental variables along the coasts of Kenya and Tanzania. We hypothesized that fish larvae abundance and community composition vary (1) across sites at a large spatial scale 10–100 km, and (2) between the NEM and SEM monsoon seasons. We also hypothesized (3) that the assumed differences in (1) and (2) are influenced by environmental factors specific to NEM and NEM seasons.

## Materials and methods

### Study area

The research was carried out in seagrass meadows in four areas in coastal East Africa (Fig. 1), including two areas in Kenya (Watamu and Diani), one area in the Tanzanian coastal mainland (Tanga) and one area on Zanzibar Island (Zanzibar). All areas are utilized by artisanal fishers for food and income^42,43^. The sampling area of Watamu is a shallow lagoon with an average depth of 5 m at high tide^43^. Tidal flushing characterizes this area, while almost the entire area dries out during low tide, except for a few deeper channels^44^. Patch reefs fringe the area on the ocean side where they break the strong waves^39^. The ebb and flood tides facilitate the exchange of materials between the lagoon and the ocean. Diani is a shallow-water coastal area with an average depth of 3.9 m at high tide^45^. As in Watamu, much of the area in Diani dries out at low tide, leaving water only in a few deeper channels. The offshore currents flow mainly northward with rates of up to 4 knots during the SEM and 3 knots during the NEM, but within the Watamu areas, the currents are weak (< 0.8 knots). The Tanga site is located in a shallow-water area with an average depth of 3.5 m at high tide^46^. The speed of water currents in this area is about 1.9 knots and a small portion of the area is exposed during low tide^42^. The sampling area at Zanzibar Island included the semi-enclosed shallow Chwaka Bay, with a total area of 50 km^2^ at spring high tide and an average depth of 3.2 m at high tide^47^, and Fumba, which is a non-estuarine open coastal area with an average depth of 10 m at high tide and a large exposed area at low tide^48^. In addition to seagrass habitats, the sampling sites of Zanzibar and Tanga comprised fringing mangroves, patches of coral reef and a variety of macroalgae.

### Sampling and sample analysis

Sampling was conducted for a minimum of three months per season at each site, repeated over two sessions (2 season cycles), except for Zanzibar, where sampling occurred throughout all 12 calendar months. Sampling took place at the sites of Watamu, Diani and Tanga from June 2019 to January 2021, and at the Zanzibar sites from January to December 2018. The sampling frequency was designed to factor in seasonality, i.e. SEM (April–October) and NEM (November–March) seasons. All sites were sampled during the daytime and at high tide (between 06:30 h and 15:00 h). Each site had two different subsites, which were selected based on accessibility and representativeness in the presence of seagrass meadows. Fish larvae were sampled using a plankton net with a removable cod end (mesh size 500 µm), a mouth diameter of 0.5 m and a length of 2.5 m, fixed with an oceanic® flowmeter in the mouth frame to estimate the filtered volume of water. The plankton net was towed horizontally (at an average depth of 1 m) for 15 min behind a motorized boat (at a speed of around 1–1.5 knots, equivalent to 2–3 km per hour) and replicated twice at each station. The net was kept at about 8 m from the rear of the boat to avoid effects of boat wake during sampling. The sampling path was random to maximise larvae capture. The fish larvae specimens were fixed with a 75% ethanol solution until further analysis. During each sampling event, in situ water parameters, including sea surface temperature (SST), salinity, pH, and dissolved oxygen (DO), were recorded in triplicate at all sites. SST and pH were measured using a multiprobe pH meter with a temperature sensor (Model STX-3). Salinity was measured using a portable refractometer (HHTEC 4-i-1) and DO concentration using an Extech 407,510 m. Opaque bottles, were used to sample surface water (500 mL) for chlorophyll *a* (chl *a*) and filtered onto 47-mm diameter Whatman GF/F filters, extracted with Ethanol and measured using spectrophotometry^49^. Surface ocean current velocity data, including eastward current velocity (Evel) and northward current velocity (Nvel), were obtained from an operational Mercator global ocean analysis and near real-time forecast system at 1/12° (0.083° × 0.083°) using the WGS84 coordinate referencing system (producing hourly mean values) with daily and monthly updated frequencies^50^. Data retrieval was done using latitude and longitude reference points for the different areas from 2019 to 2021.

In the laboratory, fish larvae were isolated from zooplankton and identified to family level using larval identification guides^51–53^. The development phase, i.e. preflexion, flexion or postflexion was assigned to each specimen, while specimens of syngnathiforms (i.e., seahorses and pipefishes) were classified as larvae or juveniles because they lack differentiated growth phases^52^. Distorted fish larvae without intact morphological features or egg yolk stage larvae that could not be identified were grouped as unidentified.

For quantifying seagrass cover and species composition, we used non-stratified sampling with randomization. Seagrass cover was estimated on a percent cover scale (0–100%) using a 50 × 50 cm quadrat and 10 random quadrats.

### Data analysis

Seagrass total cover (percentage) in all sampling sites was performed to identify the most abundant cover by species type. The percent cover per sampling site was determined by getting the total percent cover of transects divided by the number of transects used for each sampling site. PerMANOVA analysis based on Euclidean distance as a variable distance method was used to test sites and seasonal seagrass species percentage cover differences. Fish larvae that were classified as "unidentified" were only used to calculate total abundance and were excluded from the family composition analysis. Differences between sites and seasons of fish larvae total abundance, mean abundance per fish larvae family and size were calculated using two-way Analysis of variance (ANOVA) with post-hoc Tukey HSD test. Non-parametric multidimensional scaling (NMDS) with Bray–Curtis distance was used to visualize patterns of fish larvae community structure among sites and between seasons using the *vegan* 2.6–4 R package^54^. Families that drive the site distribution pattern, referred to as intrinsic variables (based on NMDS), were derived using the *envfit* function in *vegan*. PerMANOVA analysis based on Bray–Curtis distance was used to identify site and season effects on fish larvae family composition, larval stages and spawning mode with posthoc test using the *pairwise.adonis2* function^55^. For testing the assumption of dispersion homogeneity, we used PerMDISP test. Stepwise generalized linear model analysis using the MASS package was used to determine the relationship between total fish larvae abundance and environmental variables that explain the greatest seasonal variability of each site, including eastward current velocity (Evel), northward current velocity (Nvel), water temperature (SST), salinity, dissolved oxygen (DO) and chlorophyll *a* (chl *a*) by iteratively selecting or deselecting predictors based on their statistical significance. Relationships between environmental variables were tested using correlation coefficients.

## Results

### Seagrass habitat characteristics

**Figure 2:**
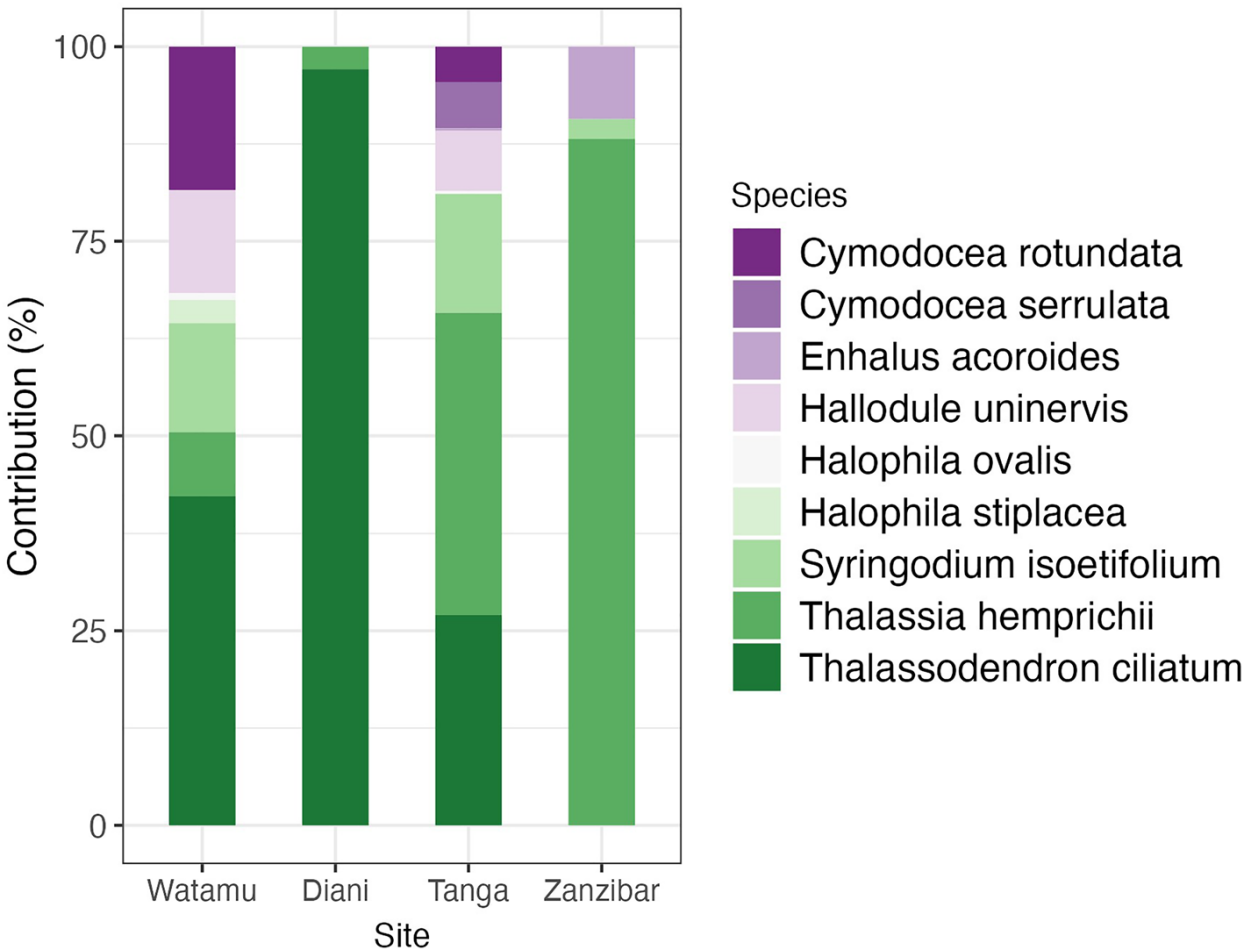
Seagrass species cover. Mean percentage cover of the seagrass species at each sampling site across the two monsoon seasons.

Seagrass species accounted for over 45% of total substrate cover in all sites. Watamu and Tanga had high seagrass species diversity with 7 and 8 species, respectively, while in Diani *Thalassodendron ciliatum* (> 96.8%) and in Zanzibar *Thalassia hemprichii* (88.1%) dominated the seagrass community (Fig. 2). These two climax species covered about 42% in Watamu (*T. ciliatum*) and > 50% in Tanga (*T. hemprichii*), indicative of an overall stable seagrass zone at all sites. Seagrass species composition differed significantly between sites (PerMANOVA, *F*_3,27_ = 58.0, *p* = 0.001) but not seasonal differences within sites (PerMANOVA, *F*_3,27_ = 0.1, *p* = 0.9).

### Fish larvae abundance and community composition across sites and monsoon seasons

**Figure 3:**
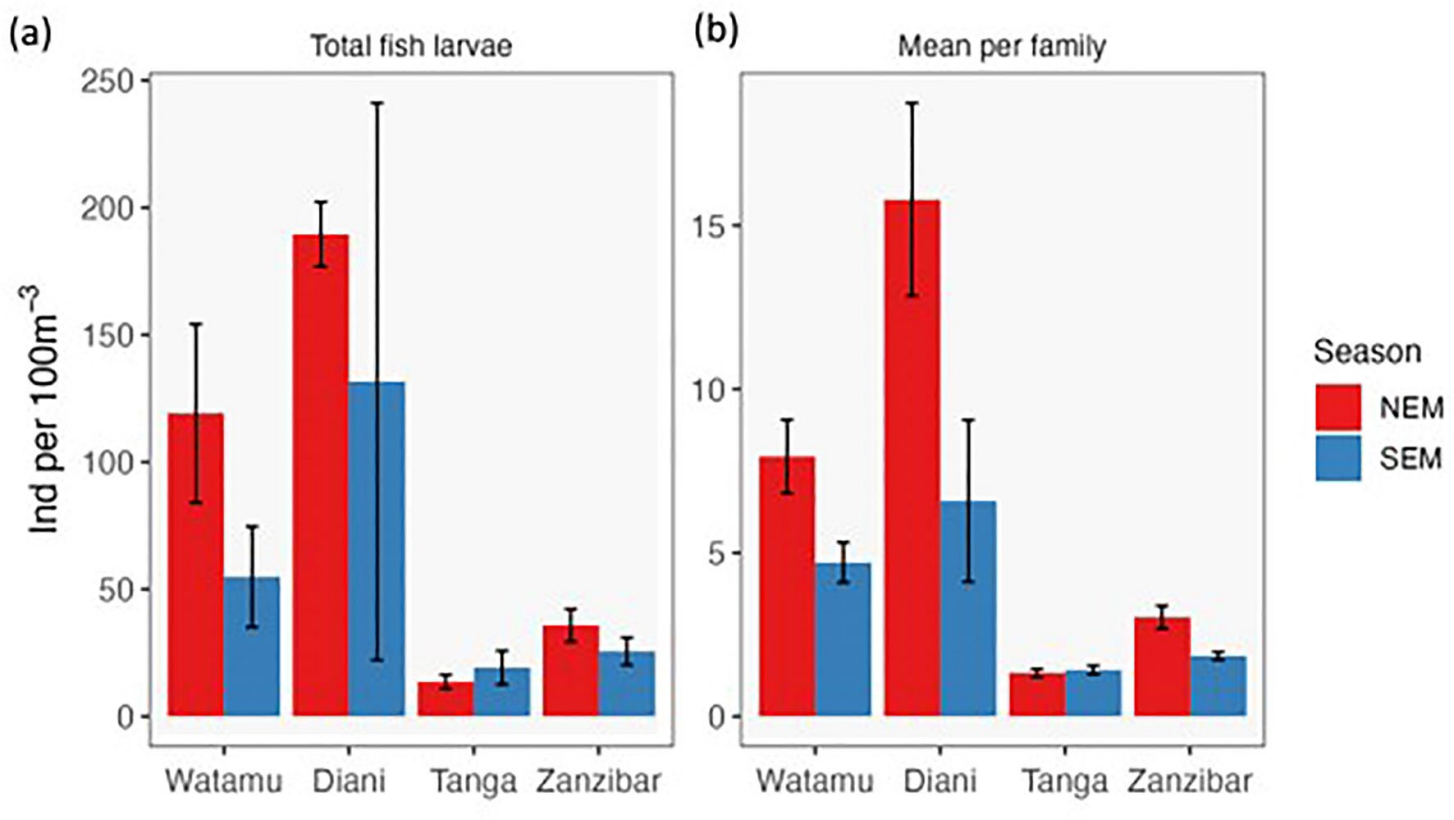
Fish larvae abundance. Abundance of (**A**) total fish larvae and (**B**) mean by familyacross the four study sites during the two monsoon seasons. Families that contributed > 2% of the total abundance are included. NEM = northeast monsoon, SEM = southeast monsoon.

During the entire study, a total of 1982 fish larvae individuals were sampled (SEM 1085, NEM 897 individuals), out of which 1941 were identified. Total fish larvae abundance over the sampling period differed between sites (ANOVA, *F*_3,27_ = 11.1, *p* < 0.001) but not seasons (*F*_1,27_ = 3.6, *p* = 0.07). Highest total fish larvae abundances were recorded in Diani with 189.5 ± 13 SE and 135.5 ± 13 SE larvae per 100m^3^ during the NEM and SEM season, respectively, followed by Watamu during the NEM season (119 ± 35 SE larvae per 100 m^3^) (Fig. 3a). Abundances were lower in Zanzibar (average of 29.8 ± 4.2 SE larvae per 100 m^3^ over the sampling period) and lowest abundances were observed in Tanga (16.3 ± 63.5 SE larvae per 100 m^3^). Mean fish larvae abundance per family ranged from 1.4 to 15 larvae per 100 m^3^, with the northernmost sites Diani and Watamu reaching the highest densities (Fig. 3b), similar to total abundance. At Diani, mean fish larvae abundance per family was significantly higher during the NEM (15 ± 2.2 SE larvae per 100 m^3^) compared to the SEM (7 ± 2.0 SE larvae per 100 m^3^) season (*p* < 0.001). Similarly, Watamu had about twice the mean fish larvae abundance during NEM than SEM (8 ± 1.0 SE vs. 4 ± 0.5 SE larvae per 100 m^3^), but here the effect of season was not significant (*p* = 0.78). Tanga had the lowest mean abundance per family (1.4 ± 0.1 SE larvae per 100 m^3^ over the sampling period), while abundance in Zanzibar were slightly higher (2.29 ± 0.2 SE larvae per 100 m^3^).

**Figure 4:**
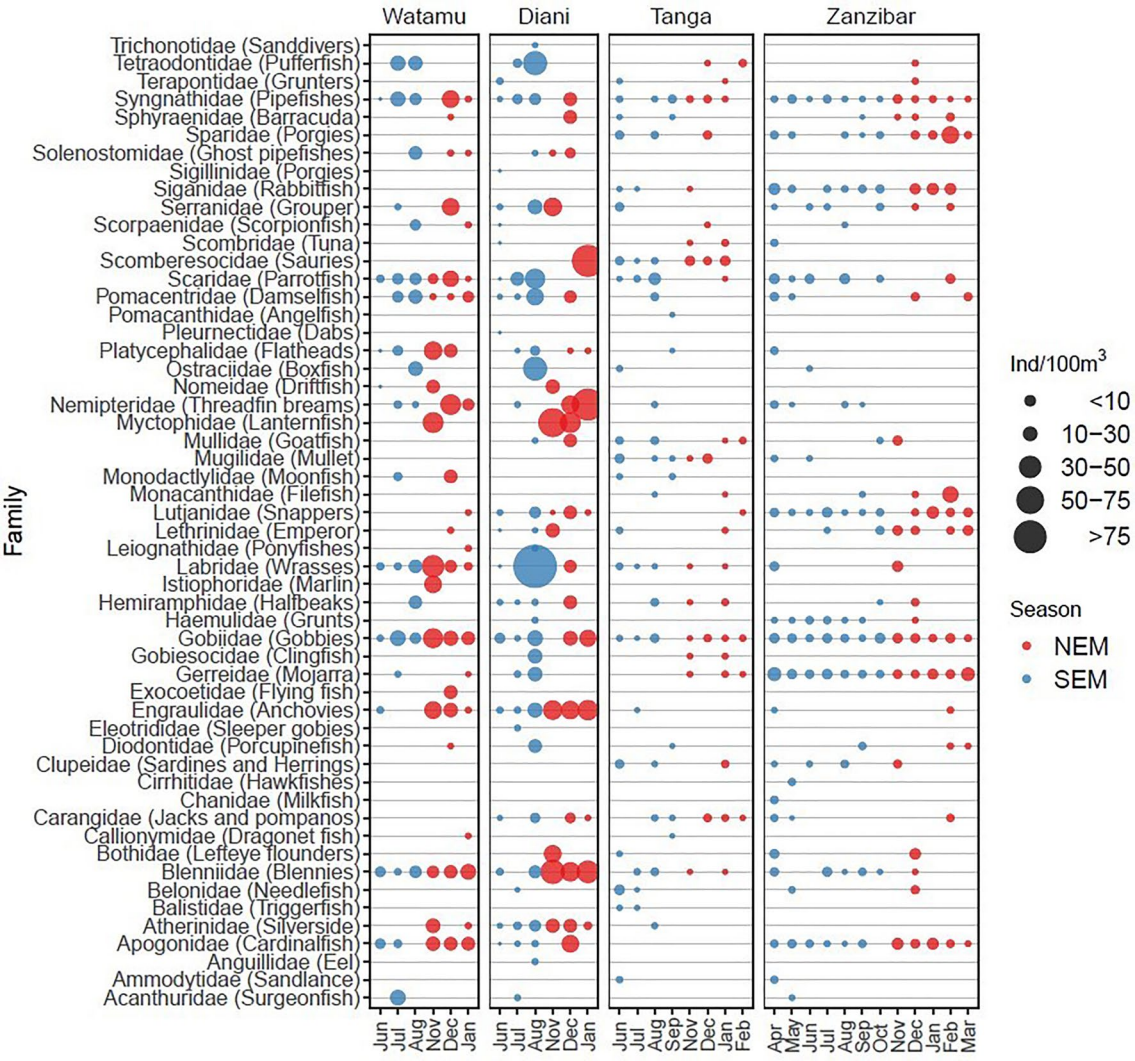
Abundance of fish larvae families. Bubble plots showing the mean abundance of fish larvae families for each sampling site over the season. NEM = northeast monsoon, SEM = southeast monsoon.

**Figure 5:**
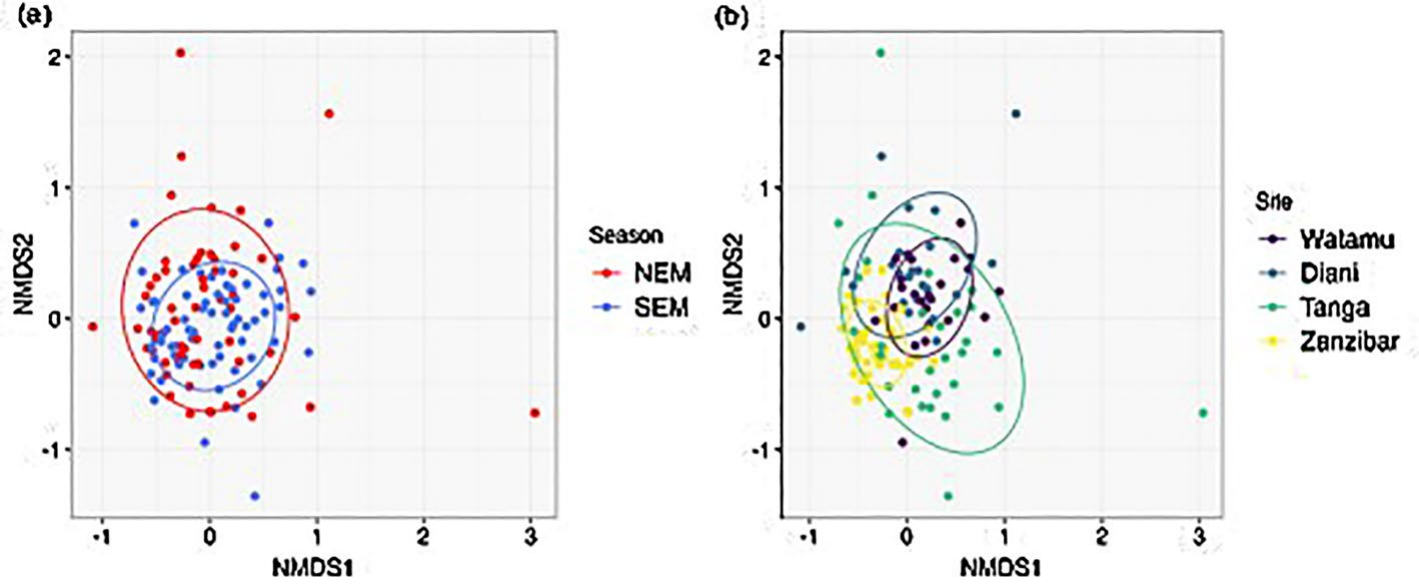
Non-parametric multidimensional scaling (nMDS) ordination of community composition of fish larvae families. Community composition across (**A**) season and (**B**) sites recorded over the entire sampling period. The stress value is 0.21. NEM = northeast monsoon, SEM = southeast monsoon.

Overall, larvae from 54 fish families were observed across the investigated areas (Fig. 4). In decreasing order of abundance, the 11 most abundant families across sites and seasons were Gobiidae, Scaridae, Syngnathidae, Gerreidae, Lutjanidae, Blenniidae, Apogonidae, Labridae, Engraulidae, Nemipteridae and Serranidae, contributing to more than 70% of total abundance. Some families were recorded only at specific sites; for example, Anguillidae, Eleotridae, Pleumectidae, Sillaginidae and Trichonotidae were exclusively detected in Diani, Istiphoridae and Cirrhitidae were only found in Watamu and Zanzibar, respectively, and Pomacanthidae and Balistidae in Tanga during SEM (Fig. 4). Fish larvae family composition was significantly different between sites (PerMANOVA, *F*_3,125_ = 7.2, *p* = 0.001) and seasons (*F*_1,125_ = 2.2, *p* = 0.01) (Fig. 5). Community composition in Zanzibar differed significantly from Diani and Tanga (*p* < 0.008), and between Watamu and Tanga (*p* = 0.021). However, as the dispersion of the fish larvae community differed among sites (PerMDISP, *F*_3,122_ = 9.9, *p* < 0.001), non-homogenous variance may interfere with these results. Fish larvae families that accounted for the difference among sites (intrinsic variables based on NMDS) were Atherinidae (R^2^ = 0.16, *p* = 0.002), Blenniidae, (R^2^ = 0.15, *p* = 0.001), Engraulididae (R^2^ = 0.10, *p* = 0.009), Gerreidae (R^2^ = 0.17, *p* = 0.002), Gobiidae (R^2^ = 0.07, *p* = 0.01), Lutjanidae (R^2^ = 0.11, *p* = 0.006), Siganidae (R^2^ = 0.06, *p* = 0.02), Syngnathidae (R^2^ = 0.15, *p* = 0.001). Atherinidae (R^2^ = 0.16, *p* = 0.002), Blenniidae, (R^2^ = 0.3, *p* = 0.001) were major families driving NEM seasonal differences, while Gerreidae (R^2^ = 0.1, *p* = 0.003), Gobiidae (R^2^ = 0.1, *p* = 0.005), Lutjanidae (R^2^ = 0.1, *p* = 0.002) were major families driving SEM seasonal differences.

### Fish larvae development stage, adult spawning mode and sizes

**Figure 6:**
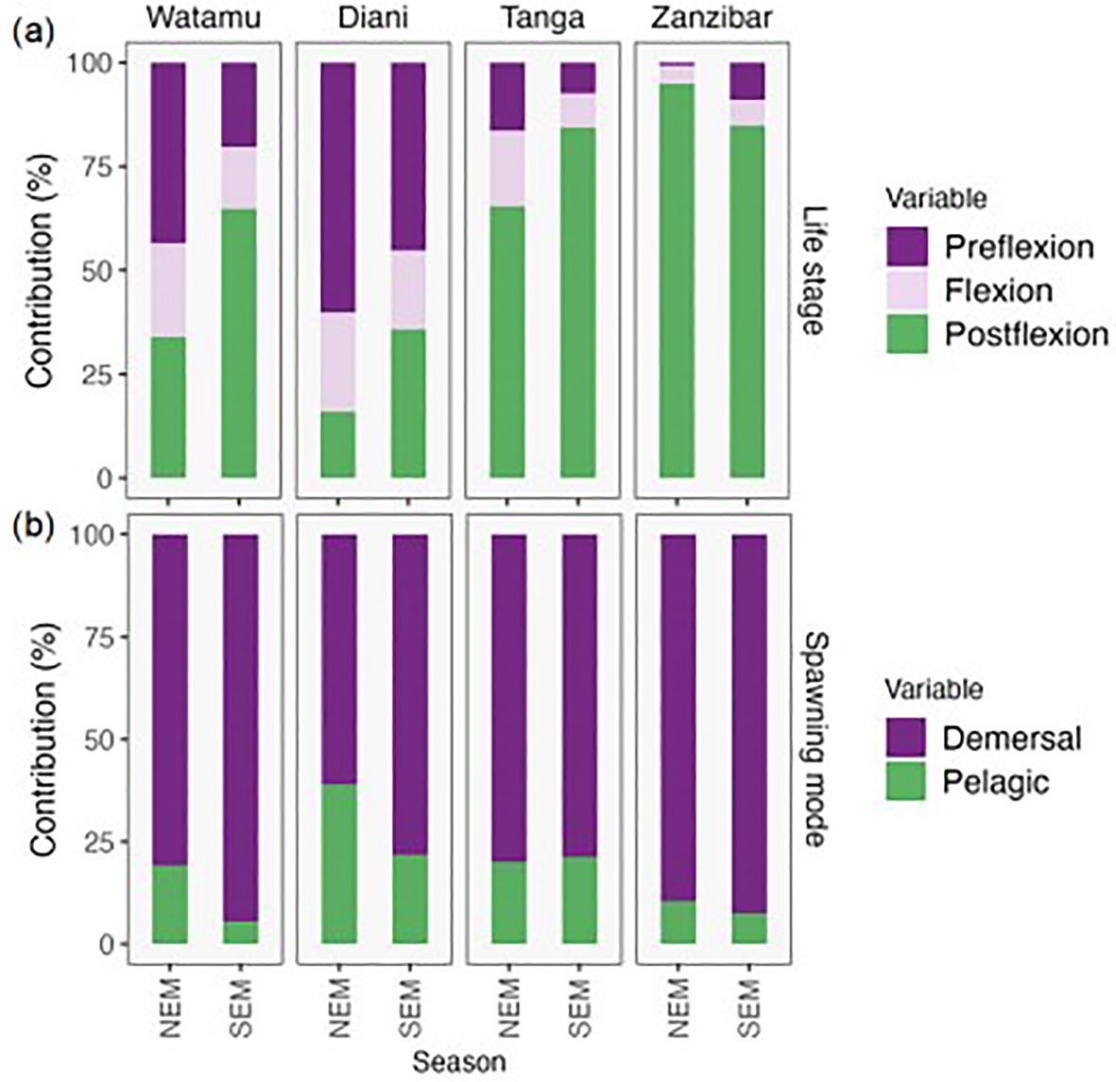
Fish larvae development stage and spawning mode. Percentage of fish larvae (**A**) developmental stage and adult (**B**) spawning mode in the different sampling sites during the NEM (northeast monsoon) and SEM (southeast monsoon) seasons.

The majority of fish larvae sampled were in the preflexion growth stage (39.1%), with slightly fewer in the postflexion (33.7%) or flexion (27%) development stages across all sites in both seasons (Fig. 6a). The percentage of fish larvae stages was similar between seasons in all sites (PerMANOVA, *F*_1,76_ = 2.8, *p* = 0.07) but significantly differed among sites (*F*_3,76_ = 20.1, *p* = 0.001). Zanzibar had the highest proportion of postflexion stage (90%), which was lower in Tanga (74.8%), Watamu (49.3%) and Diani (25.8%). Reversely, Diani (52.7%) and Watamu (31.9%) had the highest percentages of preflexion stage, which were lower in Tanga (11.9%) and Zanzibar (4.9%).

**Figure 7:**
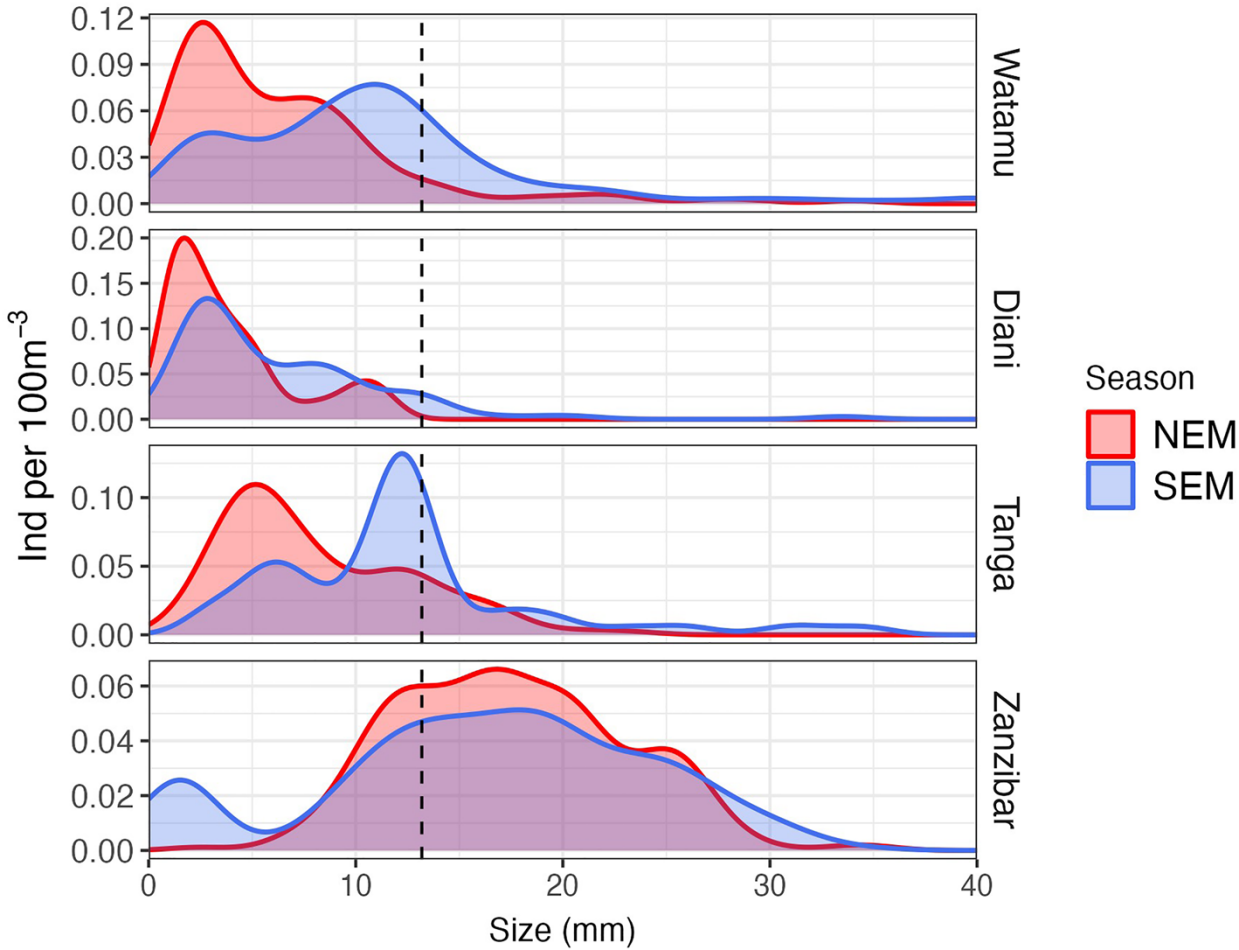
Fish larvae size. Distribution of fish larvae size at the different sampling sites during the NEM (northeast monsoon) and SEM (southeast monsoon) seasons.

Fish larvae from demersal spawning fishes predominated all sites, with an average of more than 75% of the fish specimens and significant difference between sites (PerMANOVA, F_3,31_ = 8.5, *p* > 0.002) and but not between seasons (F_1,31_ = 2.6, *p* = 0.1) (Fig. 6b). Fish larvae size ranged from 0.8 to 104 mm in total length (Fig. 7) and differed between sites (ANOVA, *F*_3,27_ = 32.3, *p* < 0.001) and seasons (*F*_1,27_ = 12.93, *p* = 0.001) in Watamu, Diani and Tanga (*p* < 0.003). Fish larvae in Zanzibar reached the largest mean size (16.9 mm, *p* < 0.001), followed by Tanga (10.0 mm) and Watamu (9.6 mm), while the lowest mean size was observed at Diani (6.4 mm, *p* < 0.008) (Fig. 7).

### Relationships between environmental conditions and fish larvae abundance

**Figure 8:**
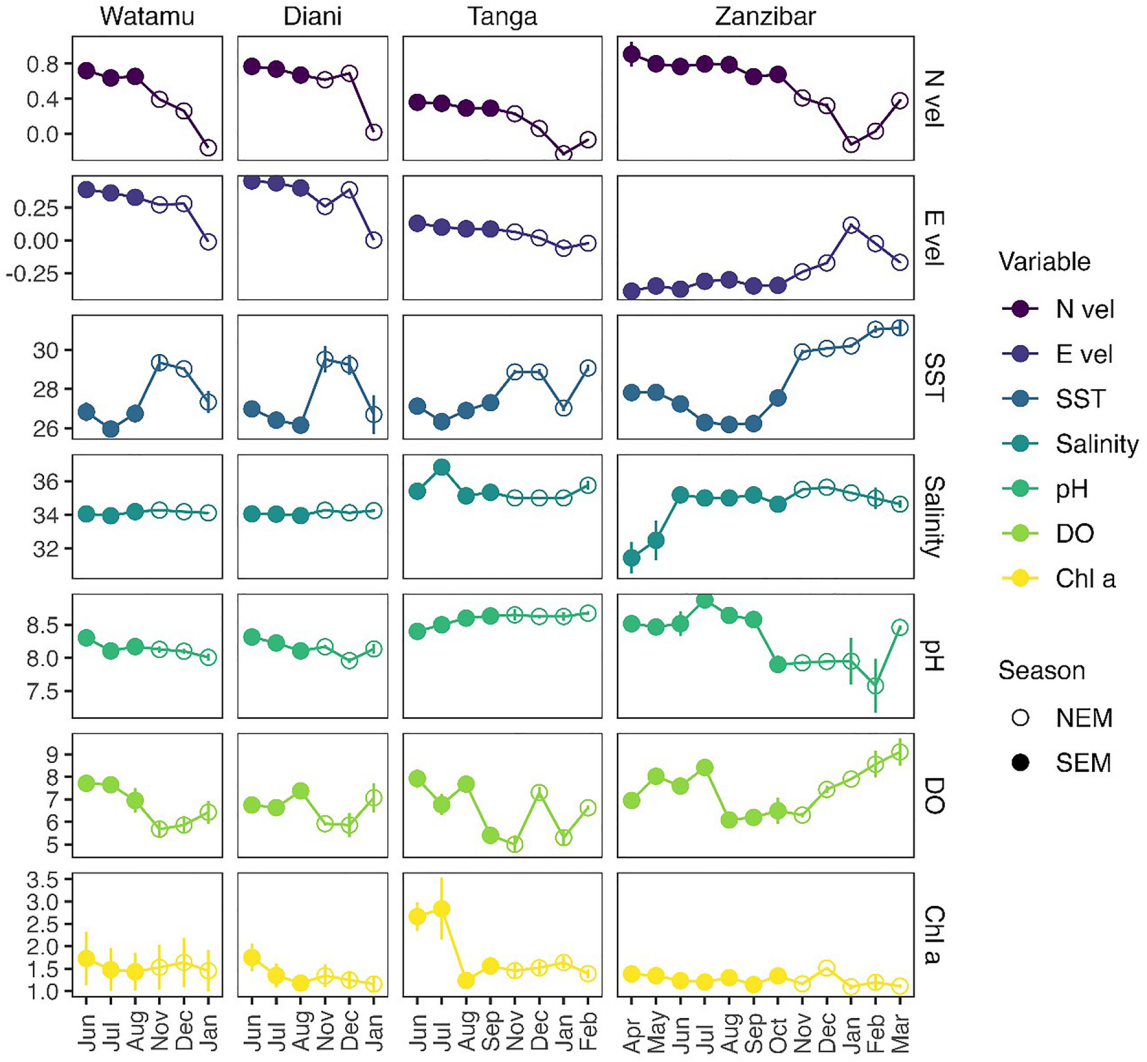
Environmental variables recorded at all sampling sites over the season. The measured environmental variables include Northward current velocity (Nvel), Eastward current velocity (Evel), sea surface temperature (SST), salinity, pH, dissolved oxygen (DO) and chlorophyll *a* (chl *a*).

The SST ranged from 25 to 31 °C during the sampling period across all sites, with the highest temperatures (slightly above 31 °C) recorded in Zanzibar and on average 1 °C higher than other sites (Fig. 8). Salinity fluctuated minimally during the whole sampling period across the different sites, varying from 33.5 to 35 ppt. Exceptions were observed in Tanga where the highest value of 37 was recorded in July and in Zanzibar where the lowest monthly salinities of 31 were observed during April and May. Water pH and DO concentration varied over the seasons between 6.5 and 9, and 4.4 and 10.9 µg L^−1^. Chl *a* concentration was in general below 2 mg L^−1^, expect Tanga had higher values in June and July (> 2.5 mg L^−1^). There was a positive correlation with Nvel and primary productivity (β = 1.04, *p* < 0.001) and DO (β = 0.07, *p* = 0.04) in Zanzibar, as well as Nvel and SST (β < 0.02, *p* < 0.03) in Watamu and Diani. A strong Evel is related to colder the water temperature (β < 0.01, *p* = 0.003) in Diani and Zanzibar (β = 0.04, *p* = 0.0003), allowing for replenishment of DO (β = 0.42).

Fish larvae occurrence in Watamu were positively influenced by potential food availability as indicated by chl *a* (GLM, LRT = 7.4, *p* = 0.001), while reduced DO concentration led to low densities of fish larvae (LRT = 7.1, *p* = 0.01) (Table 1). In Diani, total fish larvae abundances were not related to any environmental variables. In Tanga availability of DO (LRT = 1.7, *p* = 0.02) positively influenced fish larvae abundances, while SST (LRT = 2.0, *p* = 0.02) and salinity (LRT = 2.0, *p* = 0.006) show a negative effect. In Zanzibar fish larvae abundances was negatively affected by Nvel (LRT = 1.5, *p* = 0.06) and salinity (LRT = 1.3, *p* = 0.01), while food availability as indicated by chl *a* (LRT = 1.3, *p* = 0.001) and DO (LRT = 1.4, *p* = 0.001) were positively related to fish larvae abundance.

**Table 1:** GLM model output.

Site	Evel	Nvel	SST	Salinity	DO	Chl *a*
Watamu	–	–	–	–	-0.4 (0.01)	0.5 (0.002)
Diani	–	–	–	–	–	–
Tanga	–	–	− 0.1 (0.02)	− 0.2 (0.006)	0.1 (0.02)	-
Zanzibar	–	− 0.2 (0.0003)	–	− 0.1 (0.01)	0.1(0.06)	0.50 (0.001)

Results of generalized linear model (GLM) with stepwise model selection performed on fish larvae abundance at different locations and environmental predictor variables (Evel = eastward current velocity, Nvel = northward current velocity, SST = sea surface temperature, DO = dissolved oxygen, Chl*a* = chlorophyll *a*).

Values indicate regression parameters and *p*-values in parentheses.

## Discussion

We observed a high diversity of fish larvae in seagrass habitats in coastal East Africa, with more than 50 families, supporting the high species richness of this coastal region^53,56^. Fish larvae abundances were in general low at all sites without clear seasonal patterns, suggesting that most of the fish spawn year-round. The highest abundances were recorded in the northernmost sites and particularly during the NEM season. Higher total fish larvae abundances observed during the NEM season as particularly in Diani compared to the SEM season where we observe a relative weakened northward current velocity i.e. less than 0.6 m s^-1^. This may be attributed to reduced current speed and transport during the NEM season^57^, providing calmer waters suitable for spawning and more frequent local upwelling events along the coast that increase productivity and food availability^58^, as observed from the significant role played by dissolved oxygen and chlorophyll *a* variables. The occurrence of resident and transient fish larvae suggest that fish larvae composition and abundances are driven by strong hydrodynamic and tidal processes that transport fish larvae across adjacent habitats.

All the study sites were dominated by seagrass climax species, i.e. *Thalassia hemprichii* and *Thalassodendron ciliatum*. The rich biodiversity within these meadows can enhance the availability of planktonic and benthic prey, supporting the growth and development of larval fish by providing habitat, food, shelter, and nursery functions. We also find that seagrass cover did not vary between seasons, indicating that the seagrass sites are relatively stable habitats over the seasons, unless seagrass meadows are affected by changing environmental conditions, such as heat waves, eutrophication or grazing impacts^59–61^. The presence of climax species in Diani is indicative of calmer waters with relatively stable environmental and seasonal conditions favored by many fish larvae families.

Our findings showed influences of site-specific seasonal environmental variability on fish larvae abundance, especially changes in salinity, chlorophyll *a* and dissolved oxygen in Kenya and current velocities, water temperature, dissolved oxygen and salinity in Tanzania. Oxygen concentrations vary slightly between coastal habitats, especially in Watamu and Zanzibar, which are characterized by elevated primary production clearly indicating the supporting effects of localized coastal upwelling on biological production in these sites. The high proportion of preflexion life stages, especially in Diani, suggests the existence of a natural dispersal kernel and an indication that more fish completing their entire life cycle within these areas^43^ even though we did not find any relationships between fish larvae abundance and environmental variables. The Watamu area benefits from increased productivity as a result of a weak southward flow of the Somali Current during the NEM period, especially in January^62^. Here we also find that fish larvae abundance is positively affected by chlorophyll *a* concentration. During the NEM period, the EACC is also temporarily weaker and deflected northward^35,38,59^, and our study suggests that this favours the retention of fish larvae in some of the northern areas, as observed in Watamu. In general, a combination of calmer waters as observed from the weakened currents, water temperature, dissolved oxygen, chlorophyll *a* and salinity likely influence the biological processes and possibility of seeding and retention mechanisms.

In Zanzibar, velocities are more complicated, whereby Nvel is observed to be predominantly stronger during the SEM and is negatively corelated to fish larvae abundance and this causes mass water movement and turbulent mixtures as forced by strong monsoon winds. The Tanga area on the Tanzanian mainland experiences a strong EACC, i.e. a northward flowing current (1.5–2 m s^−1^), which carries nutrient-poor mid-ocean water resulting in low biological productivity within the area. However, differences in environmental characteristics, i.e. water temperature dissolved oxygen and salinity, may have a strong influence on fish larvae abundances^60,74^ due to fronts separating coastal sites from the open waters as triggered by strong coastal currents mixing over shallow waters (< 5 m). Tanzanian sites have a long continental shelf, which dries up in some areas during low tide. This zone experiences extreme temperatures (> 33 °C) and, at times, high salinity levels (> 36), especially during the NEM. Thus, the differences in fish larvae abundances among sites are likely partly influenced by the site-specific hydrodynamics that dominate in a specific region, such as variations in the direction and strength of the current velocities and affect environmental conditions^64^.

The variations in fish larvae communities across the seasons are also likely related to the spawning behaviour of the adults and fish larvae behaviour^65^. Most coastal fishes (such as Atherinidae and Blenniidae) may optimise their reproduction to high water temperatures and calmer periods associated with the NEM season, and this is observed in our case with high larvae occurring during the NEM, suggesting that seasonality influences the biological processes of fish. However, most of the fish larvae families were observed year-round suggesting a continuous adult spawning activity. Above 75% of the fish larvae were from demersal spawning families, while the rest belonged to pelagic-spawning families, indicating that many fish species move to coastal sites for spawning but migrate offshore later. This suggests that seagrass meadows are crucial for diverse pelagic and demersal spawning fish species, with demersal fish species (mostly coastal fishes) dominating in many tropical shallow-water areas, similar to other geographical areas^66–68^. The dominance of larvae from coastal fishes, e.g. gobies and blennies, reflects the existence of short distance spawning adults, whereby their larvae can disperse from a spawning ground to a small distance of up to 15 km as observed in previous studies^65^. Preflexion stage larvae of demersal fishes are likely be swept away from spawning sites into open waters by ocean currents and tidal processes, where they develop into late-stage larvae that must then find suitable habitat for settlement. The preflexion larvae survival during this period is usually low and may be influenced by feeding success and predator avoidance due to their limited swimming ability. Low abundances of individuals within the pelagic spawning Siganidae family that were sampled only in some areas in Tanzania, suggests that their larvae, especially the preflexion stage are more prone to being swept offshore^69,71,72^. Whereas the occurrence of deep-sea fishes Myctophidae and Scomberesocidae in Diani indicate retention of preflexion larvae during the NEM period. Dominant fish larval families, such as Scaridae, Gerreidae, Lutjanidae, Labridae, Engraulidae, and Serranidae, use shallow-water coastal seagrass habitats throughout their larval phases, with dispersal and aggregation in calmer areas, i.e. bays and lagoons^43^.

The majority of the fish larvae observed in this study were in the postflexion larval stage except for Diani in both seasons and Watamu during NEM, which could partly be due to the daytime sampling methodology, as the postflexion larval stage specimens migrate to deeper waters to escape the predators at the surface waters during daytime^73^. However, this can also be attributed to the active swimming behaviour of postflexion fish larvae^32^, demonstrating that fish larvae in the postflexion developmental stages specifically utilize some seagrass habitats in most of the East Africa region. The low abundance of preflexion larvae in Zanzibar in the NEM season and Tanga in the SEM season could be attributed to movement between coastal habitats and offshore areas where there is less predation, and active swimming to the coast at a larger size.

From the results we can observe fish larvae communities from resident spawners, i.e. a few kilometers or few hours from seagrass areas depending on current dynamics. The smaller size classes of preflexion and egg yok stage from families like Labridae, Scaridae and Acanthuridae form this part of community, which also happen to be year-round spawners and spawn within reefs and tidal channels. This is supported by adult fish community surveys in Watamu and Diani (unpublished data), where we identify a similar fish community of juvenile and adult stages. This suggests potential cross habitat movement of these families and within residency reproduction.

We also observe transient spawned fish larvae specifically in Diani and Zanzibar, which are likely spawned several kilometers offshore, i.e. Seranidae. Lutjanidae. While the adults are solitary in seagrass beds and coral reefs, they move offshore during spawning. This indicates that the larvae for these families are dispersed towards seagrass habitats by local currents. Siganidae, which are fringing reef spawners and are also encountered in large areas of *Thallasia* and *Enhalus* seagrass beds and extensive mangrove areas along the shore were only encountered in Tanga and Zanzibar, which have high coverage of *Thalassia* seagrass. The adults spawning takes place during NEM season, which is confirmed from our results, showing that family abundances were higher during the same season. Studies within the region identified similar fish larvae families in mangrove creeks and coral reefs with the dominant stage being preflexion larvae^17,74^. This suggests cross-habitat interactions between mangroves, seagrass and coral reef habitats and that seagrass beds can be transition nursery grounds for most coral reef fishes and appear in seagrass beds as a result of current and tidal transport processes, for refugia or feeding purposes.

Climate change within the region may have led to habitat shifts with climate warming driving the East African coastal region into an ecological desert^75,76^. Changes in environmental conditions may also affect current velocities^77^. Increasing water temperature may enhance the nutrient-poor Evel and thus reduce the survival rate of the fish larvae due to insufficient prey availability.

## Conclusions and recommendations

Here we find that site-specific seasonal environmental variability is a major factor affecting fish larvae productivity in some sites within the coast of East Africa. The general low abundances need to be confirmed with night-time sampling, but they may also indicate an overall low productivity of this region, variable environmental conditions that drive fish larvae transport processes. Low abundances may also indicate that fish larvae in seagrass habitats experience high predation pressure. Monsoon seasonality plays a partial role in determining fish larvae abundances and composition in some of the studied sites, e.g. Diani, while local hydrological and environmental conditions are the dominant environmental drivers. Our study shows that seagrass meadows are both resident and transient habitats for fish larvae that harbour various fish larvae families in the pre-settling phase that are associated with the dominant coastal fishes. The coastal fish communities are both ecologically and commercially important, suggesting that management and conservation measures should encompass seagrass habitats. This study recommends continuous monitoring of coastal habitats to detect any ecological changes or habitat shifts triggered by environmental variability due to climate change that may further reduce larval fish productivity within the East Africa coast and the Western Indian Ocean in general.

## Data Availability

Data from this study is available upon request by contacting the corresponding author.

## References

[1] Heck KLJ, Hays G, Orth RJ (2003). Critical evaluation of the nursery role hypothesis for seagrass meadows. Mar. Ecol. Prog. Ser..

[2] Madi Moussa R, Bertucci F, Jorissen H, Gache C, Waqalevu VP, Parravicini V (2020). Importance of intertidal seagrass beds as nursery area for coral reef fish juveniles (Mayotte, Indian Ocean). Reg. Stud. Mar. Sci..

[3] Alonso Aller E, Gullström M, Eveleens Maarse FKJ, Gren M, Nordlund LM, Jiddawi N (2014). Single and joint effects of regional- and local-scale variables on tropical seagrass fish assemblages. Mar Biol..

[4] Ara R, Arshad A, Amini SMN, Idris MH, Gaffar MA, Romano N (2016). Influence of habitat structure and environmental variables on larval fish assemblage in the Johor Strait, Malaysia. J. Environ. Biol..

[5] Kruse M, Taylor M, Muhando CA, Reuter H (2016). Lunar, diel, and tidal changes in fish assemblages in an East African marine reserve. Reg. Stud. Mar. Sci..

[6] Costa ACP, Garcia TM, Paiva BP, Neto ARX, de Soares MO (2020). Seagrass and rhodolith beds are important seascapes for the development of fish eggs and larvae in tropical coastal areas. Mar. Environ. Res..

[7] Olney J, Boehlert G (1988). Nearshore ichthyoplankton associated with seagrass beds in the lower Chesapeake Bay. Mar. Ecol. Prog. Ser..

[8] Russo S, Torri M, Patti B, Musco M, Masullo T, Di Natale MV (2022). Environmental conditions along tuna larval dispersion: Insights on the spawning habitat and impact on their development stages. Water.

[9] Houde ED (1994). Differences between marine and freshwater fish larvae: Implications for recruitment. ICES J. Mar. Sci..

[10] Polte P, Kotterba P, Hammer C, Gröhsler T (2014). Survival bottlenecks in the early ontogenesis of Atlantic herring (*Clupea harengus*, L.) in coastal lagoon spawning areas of the western Baltic Sea. ICES J. Mar. Sci..

[11] Doyle MJ, Ryan TA (1989). Spatial patterns in a coastal ichthyoplankton community southwest of Ireland. Rapp P-v Réun Cons int Explor Mer..

[12] Sampey A, Meekan MG, Carleton JH, McKinnon AD, McCormick MI (2004). Temporal patterns in distributions of tropical fish larvae on the North West Shelf of Australia. Mar. Freshw. Res..

[13] Beveridge MC, Thilsted SH, Phillips MJ, Metian M, Troell M, Hall SJ (2013). Meeting the food and nutrition needs of the poor: The role of fish and the opportunities and challenges emerging from. J. Fish Biol..

[14] O’Donnell JL, Beldade R, Mills SC, Williams HE, Bernardi G (2017). Life history, larval dispersal, and connectivity in coral reef fish among the Scattered Islands of the Mozambique Channel. Coral Reefs..

[15] Little MC, Reay PJ, Grove SJ (1988). Distribution gradients of ichthyoplankton in an East African mangrove creek. Estuar Coast Shelf Sci..

[16] Little MC, Reay PJ, Grove SJ (1988). The fish community of an East African mangrove creek. J. Fish Biol..

[17] Tarimo B, Winder M, Mtolera MSP, Muhando CA, Gullström M (2022). Seasonal distribution of fish larvae in mangrove-seagrass seascapes of Zanzibar (Tanzania). Sci. Rep..

[18] Somarakis S, Tsoukali S, Giannoulaki M, Schismenou E, Nikolioudakis N (2019). Spawning stock, egg production and larval survival in relation to small pelagic fish recruitment. Mar. Ecol. Prog. Ser..

[19] Whitney JL, Gove JM, McManus MA, Smith KA, Lecky J, Neubauer P (2021). Surface slicks are pelagic nurseries for diverse ocean fauna. Sci. Rep..

[20] Hutchings JA, Jones MEB (1998). Life history variation and growth rate thresholds for maturity in Atlantic salmon, Salmo salar. Can. J. Fish Aquat. Sci..

[21] China V, Holzman R (2014). Hydrodynamic starvation in first-feeding larval fishes. Proc. Natl. Acad. Sci..

[22] Houde ED (2008). Emerging from Hjort’s shadow. J. Northwest Atl. Fish Sci..

[23] Sponaugle S, Cowen RK, Shanks A, Morgan SG, Leis JM, Pineda J (2002). Predicting self-recruitment in marine populations: Biophysical correlates and mechanisms. Bull. Mar. Sci..

[24] Gary SF, Fox AD, Biastoch A, Roberts JM, Cunningham SA (2020). Larval behaviour, dispersal and population connectivity in the deep sea. Sci. Rep..

[25] Leis JM (2006). Are larvae of demersal fishes plankton or nekton?. Adv. Mar. Biol..

[26] Azeiteiro UM, Bacelar-Nicolau L, Resende P, Gonçalves F, Pereira MJ (2006). Larval fish distribution in shallow coastal waters off North Western Iberia (NE Atlantic). Estuar. Coast. Shelf Sci..

[27] Macpherson E, Raventos N (2006). Relationship between pelagic larval duration and geographic distribution of Mediterranean littoral fishes. Mar. Ecol. Prog. Ser..

[28] Besson M, Gache C, Brooker RM, Moussa RM, Waqalevu P, Lerohellec M (2017). Consistency in the supply of larval fishes among coral reefs in French Polynesia. PLoS ONE..

[29] Wolanski E, Kingsford MJ (2014). Oceanographic and behavioural assumptions in models of the fate of coral and coral reef fish larvae. J. R. Soc. Interface.

[30] Costalago D, Potter P, Pattrick P, Strydom NA (2018). Influence of environmental variables on the larval stages of anchovy, *Engraulis encrasicolus*, and sardine, *Sardinops sagax*, in Algoa Bay, South America. Environ. Biol. Fish..

[31] Macedo-Soares LCP, Freire AS, Muelbert JH (2012). Small-scale spatial and temporal variability of larval fish assemblages at an isolated oceanic island. Mar. Ecol. Prog. Ser..

[32] Guan L, Dower JF, Pepin P (2018). Characterizing spatial structures of larval fish assemblages at multiple scales in relation to environmental heterogeneity in the Strait of Georgia (British Columbia, Canada). Can. J. Fish. Aquat. Sci..

[33] Shuai F, Li X, Li Y, Li J, Yang J, Lek S (2016). Temporal patterns of larval fish occurrence in a large subtropical river. PLoS ONE..

[34] Domeier ML, Colin PL (1997). Tropical Reef Fish Spawning Aggregations: Defined and Reviewed. Bull. Mar. Sci..

[35] Leis JM, McCormick MI (2002). The Biology, Behavior, and Ecology of the Pelagic, Larval Stage of Coral Reef Fishes. Coral Reef Fishes.

[36] Council, N.R., Studies, D.E.L., Commission on Geosciences, E.R., Commission on Geosciences, E.R. Environmental Science in the Coastal Zone: Issues for Further Research [Internet]. National Academies Press; 1994. Available from: https://books.google.co.ke/books?id=n28oC9vPR6MC

[37] Gamoyo M, Obura D, Reason CJC (2019). Estimating connectivity through larval dispersal in the western Indian ocean. J. Geophys. Res. Biogeosci..

[38] Painter SC (2020). The biogeochemistry and oceanography of the East African coastal current. Prog. Oceanogr..

[39] McClanahan TR (1988). Seasonality in East Africa ’ s coastal waters. Mar. Ecol. Prog. Ser..

[40] Collins C, Reason CJC, Hermes JC (2012). Scatterometer and reanalysis wind products over the western tropical Indian Ocean. J. Geophys. Res. Oceans.

[41] Mayorga-Adame CG, Strub PT, Batchelder HP, Spitz YH (2016). Characterizing the circulation off the Kenyan-Tanzanian coast using an ocean model. J. Geophys. Res. Oceans.

[42] Wells, S., Makoloweka, S., Samoilys, M. Putting Adaptive Management into Practice : Collaborative Coastal Management in Tanga Northern Tanzania. IUCN Eastern Africa Regional Programme, (2007).

[43] Mwaluma JM, Kaunda-Arara B, Rasowo J, Osore MK, Øresland V (2011). Seasonality in fish larval assemblage structure within marine reef National Parks in coastal Kenya. Environ. Biol. Fishes..

[44] McClanahan TR, Obura D (1995). Status of kenyan coral reefs. Coast. Manag..

[45] Robinson J, Samoilys M (2013). Reef Fish Spawning Aggregations in the Western Indian Ocean: Research for Management.

[46] Samoilys, M., Kanyange, N.W. Assessing links between marine resources and coastal peoples ’ livelihoods : Perceptions from Tanga, Tanzania. IUCN report. (2008).

[47] Cederlöf U, Rydberg L, Mgendi M, Mwaipopo O (1995). Tidal exchange in a warm tropical lagoon: Chwaka Bay. Zanzibar. Ambio..

[48] Torell E, Shalli M, Francis J, Kalangahe B, Munubi R (2007). Tanzania Biodiversity Threats Assessment: Biodiversity Threats and Management Opportunities for Fumba. Bagamoyo, and Mkuranga Coastal Resources Center.

[49] Dunne RP (1999). Spectrophotometric measurement of chlorophyll pigments: a comparison of conventional monochromators and a reverse optic diode array design. Mar. Chem..

[50] Copernicus Marine Service. Global Ocean 1/12° Physics Analysis and Forecast updated Dail. Mercator Ocean International. [Internet]. 2016. Available from: 10.48670/MOI-00016

[51] Jeyaseelan MJP (1998). Manual of fish eggs and larvae from Asian mangrove waters.

[52] Leis JM, Carson-Ewart BM (2000). The Larvae of Indo-Pacific Coastal Fishes. An Identification Guide to Marine Fish Larvae.

[53] Mwaluma JM, Kaunda-Arara B, Strydom NA (2014). A Guide to Commonly Occurring Larval Stages of Fishes in Kenyan Coastal Waters.

[54] Fasham MJR (1977). A comparison of nonmetric multidimensional scaling, principal components and reciprocal averaging for the ordination of simulated coenoclines, and coenoplanes. Ecology.

[55] Arbizu M. pairwiseAdonis2 [Internet]. 2020. Available from: https://github.com/pmartinezarbizu/pairwiseAdonis

[56] Mwaluma, J.M., Okemwa, G.M., Mboga, A.M., Ngisiange, N., Winder, M., Kyewalyanga, M.S., et al. Seasonal occurrence and relative abundance of marine fish larval families over healthy and degraded seagrass beds in coastal Kenya. Diversity [Internet]. 2022; 14(9). Available from: https://www.scopus.com/inward/record.uri?eid=2-s2.0-85138671339&doi=10.3390%2fd14090730&partnerID=40&md5=d9cffdb79a6a70b03f985bc104abd485

[57] Semba M, Lumpkin R, Kimirei I, Shaghude Y, Nyandwi N (2019). Seasonal and spatial variation of surface current in the Pemba Channel Tanzania. PLoS ONE.

[58] Kyewalyanga, M.S., Peter, N., Semba, M., Mahongo, S.B. Coastal upwelling and seasonal variation in phytoplankton biomass in the Pemba Channel. *West Ind. Oc. J. Mar. Sci.* (1/2020):19–32 (2021).

[59] Burkholder JM, Tomasko DA, Touchette BW (2007). Seagrasses and eutrophication. J. Exp. Mar. Biol. Ecol..

[60] Dahl M, Deyanova D, Lyimo LD, Näslund J, Samuelsson GS, Mtolera MSP (2016). Effects of shading and simulated grazing on carbon sequestration in a tropical seagrass meadow. J. Ecol..

[61] Dahl M, McMahon K, Lavery PS, Hamilton SH, Lovelock CE, Serrano O (2023). Ranking the risk of CO_2_ emissions from seagrass soil carbon stocks under global change threats. Global Environ. Change.

[62] Benny, Peter. Variability of western Indian Ocean currents. Western Indian Ocean J. Mar. Sci. 1. 81-90 (2002).

[63] Jebri F, Jacobs ZL, Raitsos DE, Srokosz M, Painter SC, Kelly S (2020). Interannual monsoon wind variability as a key driver of East African small pelagic fisheries. Sci. Rep..

[64] Carassou L, Ponton D, Mellin C, Galzin R (2008). Predicting the structure of larval fish assemblages by a hierarchical classification of meteorological and water column forcing factors. Coral Reefs..

[65] Gray CA, Miskiewicz AG (2000). Larval fish assemblages in south-east Australian coastal waters: Seasonal and spatial structure. Estuar Coast Shelf Sci..

[66] Ara R, Arshad A, Amin SMN, Mazlan AG (2013). Temporal and spatial distribution of fish larvae in different ecological habitats. Asian J. Anim. Vet. Adv..

[67] Jaonalison H, Mahafina J, Ponton D (2016). Fish post-larvae assemblages at two contrasted coral reef habitats in southwest Madagascar. Reg. Stud. Mar. Sci..

[68] Sato N, Asahida T, Terashima H, Hurbungs MD, Ida H (2008). Species composition and dynamics of larval and juvenile fishes in the surf zone of Mauritius. Environ. Biol. Fish..

[69] Green AL, Maypa AP, Almany GR, Rhodes KL, Weeks R, Abesamis RA (2015). Larval dispersal and movement patterns of coral reef fishes, and implications for marine reserve network design. Biol. Rev..

[70] Siegel DA, Mitarai S, Costello CJ, Gaines SD, Kendall BE, Warner RR (2008). The stochastic nature of larval connectivity among nearshore marine populations. Proc. Natl. Acad. Sci. USA.

[71] Domeier ML, de Mitcheson SY, Colin PL (2012). Revisiting Spawning Aggregations: Definitions and Challenges. Reef Fish Spawning Aggregations: Biology, Research and Management.

[72] Molloy PP, Côté IM, Reynolds JD, de Mitcheson SY, Colin PL (2012). Why Spawn in Aggregations?. Reef Fish Spawning Aggregations: Biology, Research and Management.

[73] Gray CA, Kingsford MJ (2003). Variability in thermocline depth and strength, and relationships with vertical distributions of fish larvae and mesozooplankton in dynamic coastal waters. Mar. Ecol. Prog. Ser..

[74] Hedberg P, Rybak FF, Gullström M, Jiddawi NS, Winder M (2019). Fish larvae distribution among different habitats in coastal East Africa. J. Fish. Biol..

[75] Taylor SFW, Roberts MJ, Milligan B, Ncwadi R (2019). Measurement and implications of marine food security in the Western Indian Ocean: An impending crisis?. Food Secur..

[76] Wilson RJ, Sailley SF, Jacobs ZL, Kamau J, Mgeleka S, Okemwa GM (2021). Large projected reductions in marine fish biomass for Kenya and Tanzania in the absence of climate mitigation. Ocean Coast. Manag..

[77] Lo-Yat A, Simpson SD, Meekan M, Lecchini D, Martinez E, Galzin R (2011). Extreme climatic events reduce ocean productivity and larval supply in a tropical reef ecosystem: ENSO AND LARVAL RECRUITMENT. Glob. Change Biol..

[78] Redlands CESRI. ArcGIS Desktop: Release 10. 2011.

